# Recent advances in stem cell therapy: efficacy, ethics, safety concerns, and future directions focusing on neurodegenerative disorders – a review

**DOI:** 10.1097/JS9.0000000000001609

**Published:** 2024-05-15

**Authors:** Rekha Khandia, Pankaj Gurjar, Priyanka, Victoria Romashchenko, Sami A. Al-Hussain, Magdi E.A. Zaki

**Affiliations:** a Department of Biochemistry and Genetics, Barkatullah University, Bhopal, Madhya Pradesh; b Centre for Global Health Research, Saveetha Medical College and Hospital, Saveetha Institute of Medical and Technical Sciences, Chennai, Tamil Nadu; c Department of Veterinary Microbiology, College of Veterinary Science, Guru AngadDev Veterinary and Animal Sciences University (GADVASU), Rampura Phul, Bathinda, Punjab, India; d Department of Science and Engineering, Novel Global Community Educational Foundation, Hebersham, Australia; e RERC Pharmacy, RUDN University, Moscow, Russian Federation; f Department of Chemistry, Imam Mohammad Ibn Saud Islamic University (IMSIU), Riyadh, Saudi Arabia

**Keywords:** Alzheimer’s disease, amyotrophic lateral sclerosis, ethical issues related to stem cell therapy, neural cell secretome, neurodegenerative disorders, Parkinson’s disease, Huntington’s disease, stem cell therapy

## Abstract

Neurodegeneration refers to the gradual loss of neurons and extensive changes in glial cells like tau inclusions in astrocytes and oligodendrocytes, α-synuclein inclusions in oligodendrocytes and SOD1 aggregates in astrocytes along with deterioration in the motor, cognition, learning, and behavior. Common neurodegenerative disorders are Alzheimer’s disease (AD), Parkinson’s disease (PD), amyotrophic lateral sclerosis (ALS), Huntington’s disease (HD), spinocerebellar ataxia (SCA), and supranuclear palsy. There is a lack of effective treatment for neurodegenerative diseases, and scientists are putting their efforts into developing therapies against them. Stem cell therapy has emerged as a hope for neurodegenerative disorders since it is not only the damaged neurons that might be replaced, but other neuromodulators and neuroprotectors are secreted. Stem cell terminal differentiation before implantation ensures the implantation of correct cells and molecular markers like carbonic anhydrase II, CNPase (2′,3′-cyclic nucleotide 3′-phosphohydrolase), myelin basic protein (MBP), and myelin oligodendrocyte glycoprotein (MOG) elucidate the differentiation. Secretion of various growth factors like epidermal growth factor (EGF), keratinocyte growth factor (KGF), vascular endothelial growth factor-α (VEGF-α), transforming growth factor (TGF), and macrophage inflammatory protein (MIP) supports cell survival, cell proliferation, blood vessel formation, axon regeneration, and neuroglial functional connection formation at the site of degeneration. Adverse effects of stem cell therapy, like teratogenicity and differentiation in different cells other than the desired one under the influence of microenvironment, are a few key concerns. Post-transplantation improved synaptic plasticity, apoptosis inhibition, and reduction in tau-phosphorylation and amyloid beta (Aβ) production has been observed in Alzheimer’s patients. A large number of experimental, preclinical, and clinical studies have been conducted, and encouraging results have been obtained. The present review exhaustively discusses various kinds of stem cells, their usage in treating neurodegenerative disorders, limitations and challenges, and ethical issues related to stem cell therapy.

## Introduction

Highlights

- Stem cell therapy is probably the only cure for neurodegenerative disorders.
- Induced pluripotent stem cells (iPSCs), embryonic stem cells (ESCs), and various kinds of mesenchymal stem cells (MSCs), including umbilical cord MSCs, adipose tissue mesenchymal cells, and neural stem cells (NSCs) have been discussed in the present review.
- Different stem cell therapy-based approaches have been discussed for Parkinson’s disease, amyotrophic lateral sclerosis, Huntington’s disease, Alzheimer’s disease, spinocerebellar ataxia, and supranuclear palsy.
- Safety issues associated with stem cell therapy like the tendency to form teratoma and the possibility of immune rejection, are imperative to consider.
- ESCs derived from the inner cell mass of a 5-day-old to 7-day-old blastocyst raises ethical concerns, which might be addressed by chemically inducing an unfertilized embryo to develop into an embryo and using this parthenogenetic embryo for stem cell therapy.

Neurodegenerative disorders result from the progressive loss of neurons’ structure and function, leading to perturbance in motion, cognition, and learning. There is a lack of efficient therapies for the treatment of neurodegenerative disorders, which leads to economic loss for society and also leads to poor quality of life for patients and their relatives and caregivers^1^. Parkinson’s disease (PD), Alzheimer’s disease (AD), Huntington’s disease (HD), and amyotrophic lateral sclerosis (ALS) are a few names of such neurodegenerative disorders. There are various reasons for the development of neurodegenerative disorders like oxidative stress^2,3^, virus infections^4^, old age^5^ and others. Various drugs and plant extracts can be used to slow down neurodegeneration^6–10^. Apart from that, modulating signaling pathways also might help^11–13^. In an era of rapidly advancing sciences, the usage of ChatGPT might also help in suggesting solutions to treat neurodegenerative disorders^14^. The etiology of neurodegeneration is attributed to different molecular and cellular mechanisms and complex pathology. In recent eras, scientists have made attempts to treat neurodegenerative disorders, and the limited regenerative ability of brain tissue is one of the major obstacles in finding the therapy. Limited neuroregeneration is attributed to the inhibitory role of glial cells and an extracellular environment where while chondroitin sulfate proteoglycans, phosphacan, Sema3A, neurocan, MAG, and oligodendrocyte-myelin glycoprotein inhibit axonal growth and scar formation by several cytokines and growth factors prevents axonal regeneration. Nogo receptor (NgR1) inhibits neurite outgrowth, while Nogo receptor-interacting protein (LINGO-1) inhibits the differentiation of oligodendrocytes and subsequent myelination^15^. Stem cells that have the ability to grow and differentiate might serve as the solution to the problem and have the potential to repair the degeneration caused by injury, aging, or disease^16^. Stem cell or regenerative therapy enables the repairing of damaged and degenerated neural tissues. The purpose is to replace the damaged cells with the healthy ones to improve the local environment. Stem cell therapy has improved medicine tremendously by providing treatment options for numerous disorders^17^. The implications of stem cell therapy include replacing cells, secretion of growth factors through autocrine or paracrine function, or activating precursor cells^18^. In the presence of exogenous stem cells, wide varieties of bioactive products are secreted that improve neural growth, reduce apoptosis, subside inflammation, and establish synaptic connections between damaged neurons^19^.

In neurodegenerative disorders, specific cells are lost in specific brain areas. Midbrain dopaminergic (mDA) neuron death is observed in PD, while medium spiny γ-aminobutyric acid-mediated (GABAergic) neurons are lost in HD. Loss of cholinergic motor neurons in ALS and oligodendrocytes in multiple sclerosis is observed. Thus, the induction of selective differentiation is more beneficial in ameliorating neurodegenerative disorders during stem cell therapy to selectively replace lost cells of the nervous system. Multiple signals decide the fate of the cell to be differentiated. Terminal differentiation before the therapy ensures the implantation of the correct cells required for therapeutic purposes. For example, the expression of markers carbonic anhydrase II, CNPase (2′,3′-cyclic nucleotide 3′-phosphohydrolase), myelin basic protein (MBP), and myelin oligodendrocyte glycoprotein (MOG) is an indicator of differentiation of stem cells into oligodendrocytes to be used in the treatment of multiple sclerosis^20^. Another example where stem cell differentiation may be tailored to treat PD is the overexpression of Foxa2 that promotes mDA neuron formation and differentiation^21^. Thus, before stem cell transplantation, it is advisable to work on strategies that may manipulate the terminal differentiation of stem cells^22^.

The present review discusses various kinds of stem cells being used in regenerative therapies. We also discussed different clinical trials and experiments conducted on various neurodegenerative disorders. Though stem cell therapy appears to be quite promising and seems quite an attractive approach, there are many challenges related to the ethics and safety of the usage of stem cells that need to be addressed before coming to therapeutics from the bedside to the bedside.

## Types of stem cells used in therapy

**Figure 1:**
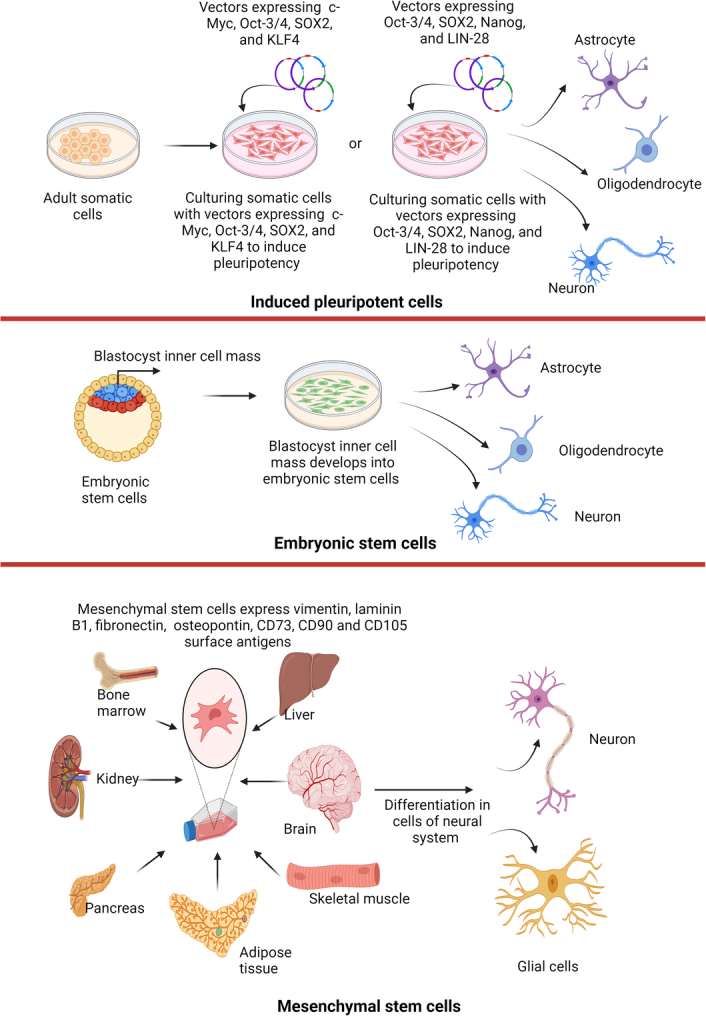
Different sources of stem cells to treat neurodegenerative disorders.

Stem cells are specialized cells which may proliferate and differentiate into various cell types. Different stem cells used in neurodegenerative disorders are given in Figure 1.

### Induced pluripotent stem cells (iPSCs)

Induced pluripotent stem cells (iPSCs) are cells generated by reprogramming animal and human differentiated cells. Here, differentiated cells are induced to dedifferentiate^23^. The imperative issue related to iPSC-based therapy is the generation of teratoma. However, during transplantation, the removal of undifferentiated cells through cell sorting measures might substantially reduce the risk of teratoma formation. In the work of Wernig *et al*.^24^, iPSCs were induced to differentiate into dopamine neurons, and post-transplantation behavior improvement was observed in a rat model of PD where mouse fibroblasts were induced for pluripotency by transfecting them with four transcription factors *Oct4, Sox2, Klf4*, and *c-Myc*.

### Embryonic stem cells (ESCs)

Embryonic stem cells (ESCs) are stem cells with pluripotency^25^ and are able to replicate indefinitely under definite conditions^26^ and are obtained from a preimplantation embryo. The greater plasticity and self-renewal ability make them clinical-grade choices for tissue replacement after injury or disease^27^. The human embryonic stem cells (hESCs) are pluripotent stem cells (PSCs) mass from preimplantation embryos^28^ expressing pluripotency markers like octamer binding transcription factor (OCT3/4), stage-specific embryonic antigen 3 (SSEA-3), stage-specific embryonic antigen-4 (SSEA-4), and T-cell receptor alpha locus (TRA-1-60 and TRA-1-81) with high telomerase activity^29^. While treating PD, post-transplantation, dopaminergic (DA) neurons from ESCs face the challenge of survival, and their viability is decreased accompanied by neuroinflammation, suggesting that neuroinflammation is the cause of reduced survival of DA neurons and supplementation of selenite during ESC transplantation therapy in PD might help reduce neuroinflammation and consequent DA death^30^.

### Mesenchymal stem cells (MSCs)

Mesenchymal stem cells (MSCs) originate from the middle and outer embryonic germ layers and, during maturation, migrate throughout the body; thus, present in a small number, it is present in a few adult tissues also^31^. These cells express vimentin, laminin B1, fibronectin, and osteopontin and boast ecto-5′-nucleotidase (CD73), Thy-1 (CD90), and endoglin (CD105) surface antigens^32^. MSCs isolated from adipose tissue especially hold promise for the development of differentiation into neuron-like cells and express distinct progenitor/mature neural markers^33^.

#### Human umbilical cord mesenchymal stem cells (hUC-MSCs)

The hUC-MSCs are able to produce a wide range of neuro-regenerative cytokines and neurotrophic factors. In the study of Xu *et al*.^34^, it has been demonstrated that transplanting hUC-MSCs into a neonatal hypoxic-ischemic encephalopathy rat model prevented damage by hypoxic-ischemic encephalopathy and improved both cognition and motor functions with decreased expression of apoptotic markers caspase-3 and Beclin-2.

#### Adipose tissue mesenchymal cells

In the work of Chung *et al*.^35^, adipose tissue-derived stem cells tested for the presence of homing cell adhesion molecules (CD44), CD73, CD90, CD105, and human leukocyte antigen (HLA)-ABC. Transient global cerebral ischemia-induced hippocampal neuronal death was reduced by administrating adipose tissue-derived grafted MSCs, and a significant reduction in endothelial damage was observed. It poses a neuroprotective impact, possibly through stabilizing the disrupted blood–brain barrier and, therefore, preventing inflammatory cell influx.

#### Neural stem cells (NSCs)

**Figure 2:**
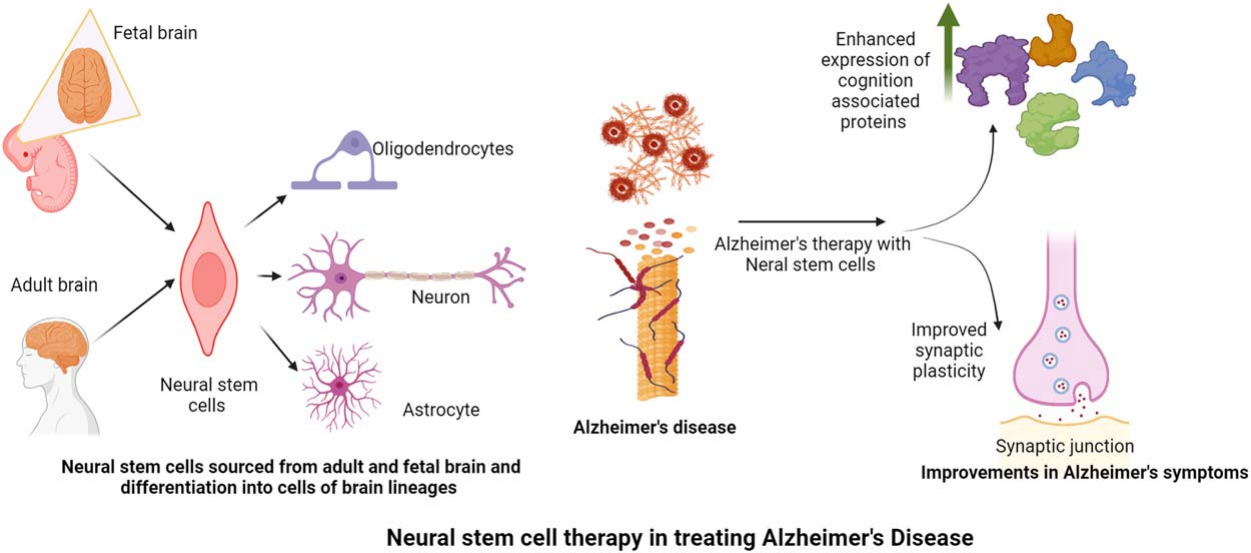
Role of neural stem cells in ameliorating the symptoms of Alzheimer’s disease.

**Figure 3:**
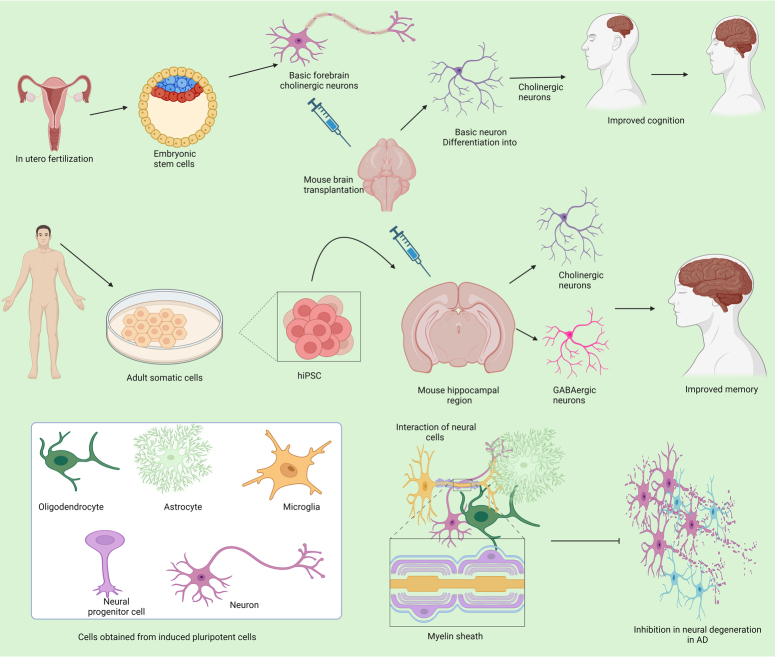
Stem cells from various sources in inhibition of Alzheimer’s disease.

The NSCs are specialized neural cells present in brain tissues and have lesser potential to regenerate^36^, and these differentiate in limited types of cells of brain lineages like oligodendrocytes, neurons, and astrocytes^37^. The NSCs might be derived from different brain parts from both the fetus and the patients undergoing brain surgical procedures^38^. Human NSCs have been proven beneficial due to synaptic plasticity and ameliorating AD symptoms through upregulating the expression of multiple cognition-related proteins (Fig. 2)^39^. Intra-hippocampal transplantation of human CNS stem cells (HuCNS-SC) transplantation exhibited their migration and differentiation into immature neurons and glia with increased synapsis and improvement into symptoms of AD was visible not due to reduction in Aβ or tau pathology, but due to enhanced endogenous synaptogenesis^40^ and thus human NSCs are a potential source of therapeutic grade stem cells to treat the neurodegenerative disorder. In the phase I clinical trial to test the efficacy and safety of fetal human NSCs, the cell transplantation into the anterior horns of the spinal cord showed no progression of ALS and a transient improvement in symptoms^41^. Contrary to ESCs, NSCs are genetically more stable and less teratogenic, and their less self-regenerative properties can be addressed using genetic manipulations and the usage of mitogens. Still, there are challenges related to the immunogenic intolerance of allogenic NSCs, limited resources, difficulties in isolation, and expansion with associated religious concerns^38^. Stem cells (obtained from various sources like embryonic, adult somatic cells, or from induced pluripotent cells) mediated inhibition of AD is given in Figure 3.

##### 2.3.3.1 NSC secretome and its role in brain repair

The potential of NSCs in neuroprotection is not only limited to *de-novo* generation of graft-derived neurons and glial cells but NSCs have been found to provide support to damaged cells through resisting further damage, repairing, regeneration of damaged tissue and reducing in inflammation and inflammatory cell influx^42^. Stem cells secrete various growth factors like epidermal growth factor (EGF), keratinocyte growth factor (KGF), vascular endothelial growth factor-α (VEGF-α), transforming growth factor (TGF) and macrophage inflammatory protein (MIP) to recruit macrophages and endothelial cells to injury sites to promote support cell survival, cell proliferation, axon regeneration, and blood vessel formation^43^ and regeneration of lost tissues in neurodegenerative disorders^44^. During spinal cord injury, transplanting GABAergic neural progenitor cells has been shown to attenuate neuropathic pain and improve locomotor load in rat models^45^. After neuronal loss, not only are the lost neurons replaced by stem cell therapy, but they also help establish neuroglial functional connections lost during the neurodegenerative pathology^46^. There are several challenges associated with the transplantation of NSCs. The key challenges are low survival and irrational differentiation. The neuroinflammatory conditions and autophagy influence the differentiation of neural cells and might adversely affect functional recovery^47^. Induction of expression of selective markers for specific cell differentiation like NSCs form a secretome consisting of biological molecules that influence in a paracrine manner, and imperative ones are as follows.

- Prostaglandins
  The NSCs release various cell adhesion molecules (e.g. CD44), integrins (e.g. a4b1), and chemokine receptors (e.g. CCR2, CX3CR1, and CXCR4) in order to reach the inflamed tissue^48^. Established cell-to-cell interaction induces Fas-mediated apoptosis of Th1 and Th17 T cells. G-protein coupled receptor 91 (GPR91) works as an autocrine and paracrine sensor, activating the production of anti-inflammatory prostaglandin E2 (PGE2) that acts as a switch from pro-inflammatory to anti-inflammatory response and downregulates IL-1b production^49^.
- miRNAs
  The immunomodulatory effects of microRNA (miRNA) in neural stem cells (NSCs) are largely unexplored. NSCs within the subventricular zone generate neuroblasts that differentiate into granule and periglomerular interneurons^50^. The role of miR-124 is evident in the process of proliferation and differentiation of NSC by inactivating the Notch pathway^51^ and cleaving of polypyrimidine tract binding protein (PTBP1), a repressor of neuron-specific splicing^52^ and knocking down of endogenous miR-124 results in delay in neuroregeneration^53^. MicroRNA-124-loaded nanoparticles have been demonstrated to increase the survival and neuronal differentiation of NSCs *in vitro* ^54^. The evidence clearly demonstrates the specific roles of miRNA in the NSC secretome.
  Another miRNA, Let-7b, is expressed in mammalian brains. Its expression is increased during neural differentiation, and the process is mediated through the stem cell regulator orphan nuclear receptor (TLX) and the cell cycle regulator cyclin D1. Therefore, using Let-7b might help differentiate NSCs during stem cell therapy in neurodegenerative disorders^55^. miRNAs miR-17 and miR-7a determine the neuronal fate^56^. Comprehensive miRNA sequencing profiling of pluripotent human embryonic stem cells exhibited differential expression of miRNAs during neural cell fate determination, and miR-124, miR-9, and miR-219-a-2 were significantly upregulated only in terminally differentiating NSCs^57^.
- Cytokines

Damaged cells secrete cytokines including interleukins (IL-1ß, IL-12), tumor necrosis factor alpha (TNFα), and nitric oxide synthase (iNOS), exacerbating the neurotoxicity and, in response, microglia adapts ameboid morphology and release neuroprotective and anti-inflammatory factors such as IL-10, transforming growth factor-beta (TGFβ), and the mannose receptor (CD206)^58^.

## Stem cell therapy for treatment of neurodegenerative disorders

Stem cell therapy is the only modality that can cure neurodegenerative disorders^59^. In experiments conducted in animal models^38,59–61^ and in some preclinical and clinical studies^59,62^, structural and functional improvements have been observed in neurodegenerative disorders. However, long-term safety issues are imperative questions that need to be answered.

### Stem cell therapy in PD

PD is characterized by motor dysfunctions like bradykinesia, rigidity, rest tremor, and postural instability^63^. Dopaminergic neurons of the substantia nigra pars compacta are selectively lost, and as a result, dopamine levels are reduced. Other non-motor symptoms include sleep disturbance, anosmia, neuropsychiatric features, and cognitive decline^64^. Presently, anti-PD therapy generally involves dopamine replacements and is able to provide only symptomatic relief^11^. Since neurodegeneration in specific parts of the brain is responsible for PD symptoms^65–67^, scientists have great interest in regenerative therapies, and attempts are being made to replenish degenerated neurons with the stem-cell-derived equivalents obtained from ESCs, NSCs, and induced pluripotent cells (iPSCs)^68^. In an open-label study, intracerebral transplantation of autologous bone marrow-derived MSCs and subsequent follow-up for 12 months exhibited no adverse effects. However, the study neither confirmed the site of transplantation in the brain nor confirmed whether, under the brain environment, the bone marrow MSCs potentially differentiated into neural cells or dedifferentiated into PSCs, which could pose detrimental effects in the future^69^. In the presence of stromal cell-derived inducing activity and the absence of bone morphogenic protein-4, ESCs might differentiate into neural precursors and neurons^70^. Such progenitors expanded into neurospheres encompassing DA neurons and transplantation of such cells into a primate model for PD, reversed the symptoms of Parkinson’s and showed the therapeutic efficacy of stem cells in treating PD^71^.

**Figure 4:**
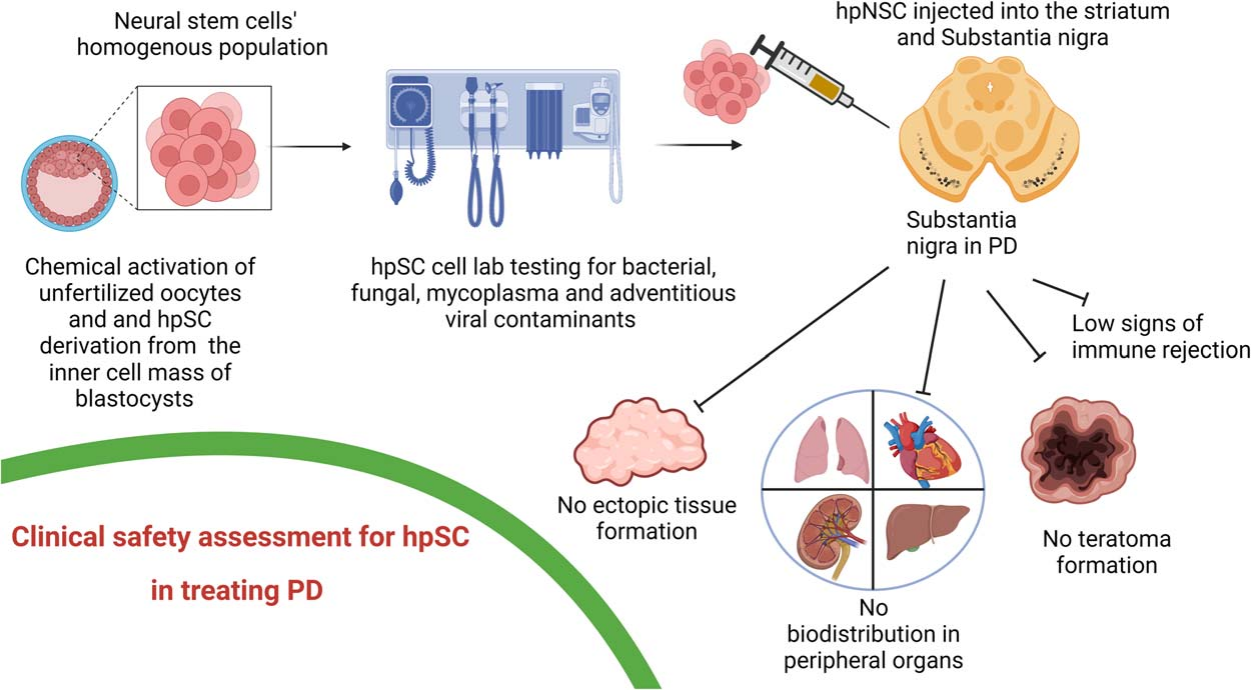
Schematic presentation of clinical safety assessment of human parthenogenetic derived neural stem cells (hpNSC) in treatment of Parkinson’s disease.

The human PSCs based open-label, dose-escalating, phase I study was conducted by Garitaonandia *et al*.^72^ for assessment of safety and tolerability of human parthenogenetic derived neural stem cells (hpNSCs) which were injected into the striatum and substantia nigra of PD patients. In the experiment, human parthenogenetic stem cells (hPSCs) can be obtained from the chemical activation of unfertilized oocytes, thus bypassing the human ethical concerns since no killing of the human embryo is required (Fig. 4).

### Stem cell therapy in ALS

ALS is the gradual loss of nerve cells in the brain, spinal cord, and muscle cells. In ALS, both upper and lower motor neurons are involved, and difficulty in swallowing, speech, and respiratory muscles is involved^73^, eventually leading to various disabilities and death. It is the most devastating neurodegenerative disorder, taking the life of a patient within 5 years of diagnosis^74^. In improving the pathophysiology of ALS, only two licensed therapeutic options, riluzole and edaravone, have been proven to be less effective^75^. Riluzole is a drug that has shown promise in reducing ALS-mediated mortality, and up to 35% reduction in mortality has been observed in a clinical trial^76^. Attempts are being made to use anti-sense oligonucleotide targeting mutations in *SOD1* or *C9orf72* genes, which are also underway. However, not much success is observed in the treatment of ALS^77^. Considering the stem cell therapy, in the experiment conducted in the SOD1 (G93A) rat model, transplantation of lineage-restricted astrocyte precursors, called glial-restricted precursors (GRPs), resulted in GRPs differentiation into astrocytes and reduction in microgliosis. Also, extended survival with reduced motor neuron loss^78^ was observed. In 2012, in a clinical trial of 18 ALS patients, the safety of stem cell transplantation was observed by implanting *ex-vivo* expanded bone marrow-derived MSCs, and it was found safe and well tolerated in ALS patients. In the study of Zhu and Lu^74^, transplanted NSCs or NPCs exhibited excellent survival and regenerated axonal connections with the host connectivity, including motor axons in the ventral root. This reciprocal connection is essential to re-establish the voluntary motor control of muscles^79^. In the phase I trial encompassing 27 patients, intrathecal administration of adipose tissue-derived stem cells was well tolerated with fewer side effects, which resolved over the period. The autopsy of four dead patients exhibited no sign of tumor development, and anecdotal mild, temporary subjective clinical improvement was observed^80^. Glial cell line-derived neurotrophic factor (GDNF) is a potent growth factor and an imperative survival factor for dopaminergic, motoneurons, and noradrenergic neurons. It has protective roles for neuromuscular junctions^81^. Considering the protective roles of GDNF, GDNF-secreting engineered human cortical-derived neural progenitor cells were transplanted into SOD1G93A ALS rat and Cynomolgus macaques cortex. In both cases, robust expression of GDNF was observed. In the ALS model for rats, disease pathology was delayed, and extended survival of animals was achieved^82^. Stem cell therapy for ALS is still in its infancy, so the appropriate origin of stem cells with the suitable site for injection of stem cells and the dose need to be meticulously optimized.

### Huntington disease (HD)

HD is a progressive, fatal degenerative disorder where CAG repeats are present^83–85^ in the Huntingtin gene (ht), and mutations in the gene result in the loss of medium spiny neurons^86^, culminating in motor, cognitive, and emotional deficits^87,88^. Neurodegenerative research is less in the case of HD, and fewer trials have been conducted at the clinical level^89,90^. Still, evidence shows promising beneficial roles of NSCs in ameliorating phenotypic changes during HD^91^. Genetically engineered bone marrow MSCs, obtained from femur bone to express brain-derived neurotrophic factor (BDNF) or nerve growth factor (NGF) were transplanted intrastriatally and created an environment that reduced the speed of neurodegeneration and behavioral changes in YAC 128 transgenic mice. Long-term safety and efficacy determination are required for this approach to be inculcated into the clinical utility^92^. Dental pulp stem cells (DPSCs) are considered adult stem cells with pluripotent differentiation abilities, can be isolated from a person of any age, and are true autologous stem cells. DPSCs isolated from transgenic monkeys expressing both mutant ht and green fluorescent protein (GFP) genes exhibited properties like self-renewal, multipotent differentiation capabilities, expression of stemness and differentiation markers, and cell surface antigen profile and thus might be helpful in stem cell-based therapies^93^. Implantation of bone marrow-derived human multipotent stromal cells (hMSCs) into HD rat transgenic mice (N171-82Q) model revealed the disappearance of hMSCs within 3–5 days; however, these transplanted cells stimulated the proliferation and differentiation of endogenous NSCs in mice and improved the conditions of HD. However, parameters like the dose of cells, route of administration, and other safety concerns should be addressed and optimized^94^.

### Alzheimer’s disease (AD)

AD is evidenced by senile plaques and neurofibrillary tangles^95^ and is the most common type of dementia^96,97^, and neurodegeneration is evident^98,99^. To prevent the disease progression, a multifaceted approach is required to promote cell survival and replacement for lost neurons^100^. NSCs can differentiate into neurons and glial cells, and these are present in the cortical subventricular zone and dentate subgranular zone. Neurogenesis has been identified in the hippocampal region of persons aged 100 years^101^. During the AD progression, the adult hippocampal neurogenesis is prevented, and this exogenous stem cell therapy is effective in the ‘bystander effect’ where paracrine functions like secretion of different metabolites and neurotrophic factors help in rejuvenating and maintaining the host niche^100^. Human induced pluripotent stem cells (hiPSC), when induced into the hippocampal region of AD mice model of dementia, the presence and proliferation of hiPSC resulted in differentiation into cholinergic and GABAergic neurons and culminated into improved spatial memory^102^. ESCs were modified to differentiate into basic forebrain cholinergic neurons (BFCN) progenitors and transplanted into AD rodents’ brains. Two months after transplantation, the progenitors were differentiated into mature cholinergic neurons with similar functional integrity to the host cholinergic system and improved cognitive deficits^103^. Human NSCs transplanted into the intra-cerebroventricular in transgenic AD mice models showed migration of engrafted cells toward multiple affected brain regions. The stem cell transplantation exhibited improved synaptic plasticity, apoptosis inhibition, and reduction in tau-phosphorylation and amyloid beta (Aβ) production^12^. Moreover, safety and efficacy are the significant concerns related to stem cell-based therapies against treating AD.

### Spinocerebellar ataxia (SCAs)

SCAs is a heterogeneous genetic disorder^104,105^. In the *ATXN3* genes, increased CAG sequence repeats lead to the expanded polyglutamine, resulting in several SCAs. Currently, only taltirelin, a thyrotropin-releasing hormone (TRH) analog, has been approved as a palliative treatment for alleviating the symptoms of SCAs^106,107^. In the experiment of Han *et al*.^108^, patients’ urine-derived iPSCs were subjected to differentiate into neurons and co-culture of these MSCs inhibited intracellular mutated ataxin-3 proteins and had ameliorating effects on SCAs. In the phase I/IIa clinical study, allogeneic adipose tissue-derived MSCs obtained from healthy donors were intravenously administered. During the one-year follow-up, no adverse effect indicated that the treatment was tolerable, safe, and exhibited therapeutic potential^109^. It is warranted to have a dose- and frequency-finding design and evaluation of proteins or miRNA biomarkers in biological fluids to correlate and reduce the severity of SCAs.

### Supranuclear palsy

Supranuclear palsy is characterized by progressive neurodegeneration^110,111^, and, as a result, an exhibition of Parkinsonism is there. A 71-year-old Korean male was subjected to stem cell therapy as a case study. The stem cells were obtained from adipose tissue and given as intravenous and intrathecal infusions. The results were exciting, and apparently, there were no adverse effects, excluding intermittent mild fever and transient high blood pressure; otherwise, the treatment was well tolerated and delayed the neurodegeneration and progression of supranuclear palsy^112^. Table 1 reveals information regarding various clinical trials conducted using stem cell therapy.

**Table 1:** Details of clinical trials conducted with stem cell therapy to treat neurodegenerative disorders.

Name of disease	Type of cell	Source of stem cells	Route of administration	Purpose/expected outcome/result /interpretation	Clinical trial in phase	Participant numbers	Study location	NCT number
Amyotrophic lateral sclerosis (ALS)	Mesenchymal stem cells (MSCs) from adipose tissue	Autologous	Intravenous (03 doses)	Evaluation of the safety of administration of 3 doses of intravenous adipose tissue-derived MSCs	Phase I/II Clinical Trial	52	Spain	NCT02290886
	MSCs	Autologous	Intraspinal	Evaluation of the safety of intraspinal delivery of MSCs in a dose-escalation study	Phase I	27	United States	NCT01609283
	Bone marrow-derived MSCs	Allogenic	Intrathecal	Safety studies of HLA – haplo-matched allogenic bone marrow-derived stem cells	Phase I	06	Republic of Korea	NCT01758510
	Adipose tissue-derived stem cells	Autologous	Intravenous and intracerebral	Assessment of the efficacy of brain transplants of autologous adipose tissue-derived stem cells	Phase I	01	Taiwan	NCT02383654
	Bone marrow-derived MSCs	Autologous	Intrathecal	A possible clinical benefit that lasted safely for at least 6 months through switching from pro-inflammatory to anti-inflammatory conditions	Phase I Phase II	72	Republic of Korea	NCT01363401
	Cultured bone marrow-derived MSCs secreting neurotrophic factors (MSC-NTF)	Autologous	Multiple intramuscular injections at 24 separate sites + single intrathecal injection into the CSF	87% of patients responded to the therapy to ALS Functional Rating Scale-Revised/forced vital capacity exhibiting at least 25% improvement at 6 months after treatment	Phase II	14	Israel	NCT01777646
	Bone marrow-derived MSCs	Autologous	Intrathecal	>25% improvement in the slope of progression of Amyotrophic Lateral Sclerosis Functional Rating Scale-Revised (ALSFRS-R)	Phase I Phase II	20	Hadassah Medical Organization	NCT04821479
	MSCs	Autologous	Intraspinal (at subarachnoid space)	To monitor blood pressure, temperature and pain score after MSC transplantation	Phase I	01	United States	NCT01142856
	Neurotrophic factors-secreting bone marrow-derived MSCs	Autologous	Sites of damage, the spinal cord and the muscles	Post-transplantation an increase of ≥1.5 points/month in ALSFRS-R slope, a significant increase in CSF neurotrophic factors, and a decrease in CSF inflammatory biomarkers	Phase II	48	United States	NCT02017912
	Human neural progenitor cells expressing GDNF	Allogenic	Lumbar region	Graft survival and production of glial cell line-derived neurotrophic factor (GDNF) is observed	Phase 1/2a,	18	United States	NCT02943850
	Cultured bone marrow-derived MSCs secreting neurotrophic factors	Autologous	24 intramuscular injections (IM) on the biceps and triceps muscles	87% of patients responded to either ALS Functional Rating Scale-Revised or forced vital capacity with at least 25% improvement in the slope of progression	Phase 1/2 trial	12	Israel	NCT01051882
Alzheimer’s disease	Human MSCs	Allogeneic	Intravenous (~100 million cells)	Evaluation of the presence of adverse effects related to the study intervention like patient hospitalization, persistent or significant disability/incapacity, clinically asymptomatic brain microhemorrhages or death	Phase I	06	United States	NCT04040348
Huntington’s disease	Product Cellavita HD	Allogenic	Intravenous (03)	Restoration of the BDNF, DARPP32, and D2R expression and resulting neuroprotection and neurogenesis	Phase II	35	Brazil	NCT03252535
	Somatic cells derived induced pluripotent stem cells	Allogenic	–		Recruiting	120	Israel	NCT00874783
Parkinson’s disease	Stem cells from placental amnion donated after cesarean section in healthy women	Allogenic	Lateral ventricle	Evaluation of the presence of adverse effects like whether the treatment is fatal, life-threatening, requires in-patient hospitalization or prolongation of existing hospitalization, results in persistent or significant disability/incapacity, congenital anomaly/birth defect, or other significant medical hazard	Early Phase I	03	China	NCT04414813
	Bone marrow-derived MSCs	Allogeneic	Infusion	Selection of the safest and most effective number of repeat doses of allogeneic MSC infusions	Phase II	45	United States	NCT04506073
	Bone marrow-derived MSCs	Allogeneic	Intravenous	Post-treatment, no serious adverse reactions related to the infusion and no responses to donor-specific human leukocyte antigens	Phase 1	20	United States	NCT02611167
	Platelet rich plasma (PRP) + peripheral blood-derived very small embryonic-like (PBD-VSEL) stem cells	Autologous	Intravenous (at four acupuncture points)	Evaluation of the improvement in the Unified Parkinson’s Disease Rating Scale (UPDRS), Hospital Anxiety and Depression Scale (HADS), and self-report Parkinson’s Disease Questionnaire-39	NA	30	Pakistan	NCT06142981
	Hope Biosciences – adipose-derived MSCs	Autologous	Intravenous	Testing the efficacy and safety of multiple HB-adMSCs vs. Placebo	Phase II	24	United States	NCT04928287
Progressive supranuclear palsy	Bone marrow-derived MSCs	Autologous	Intravenous and intranasal	Assessment of neurologic function post-treatment Neurology Quality of Life encompassing activities like Communication, Anxiety, Depression, Emotional and Behavioral Dyscontrol, Mobility, Sleep Disturbance, and Cognitive Function	Recruiting	500	UAE	NCT02795052
Ataxia	Adipose-derived MSCs	Allogenous	Intravenous	Assessment of Scale for the Assessment and Rating of Ataxia (SARA) score	Phase I Phase II	07	Taiwan	NCT01649687
	Stemchymal	Allogenous	Intravenous	SARA score and safety assessment by examining vital signs, blood biochemistry tests, complete blood count, immunoactivity assay, urinalysis, and magnetic resonance imaging	Phase II	56	Taiwan	NCT02540655

## Issues related to poor differentiation during stem cell therapy

ESCs have been generated after processing the inner blastocyst of the fertilized embryo, and various differentiation protocols have been used to differentiate cells into neural cells. hESCs are revealed to produce mDA neurons, which robustly survived for several months in cell culture, and functions were demonstrated in mice rat and primate models^113^. Dopamine production depends on the rate-limiting enzyme tyrosine hydroxylase (TH), and expression of TH can be induced; however, the yields of TH-positive cells are highly variable. Still, these cells showed recovery in motor function in animal models^114^. Different dopaminergic cell sources where neurons produce dopamine have been used. However, only a few were reported to produce authentic mDA nigral neurons since these were not expressing LIM homeobox transcription factor 1 alpha (LMX1A) and Forkhead box protein A2 (FOXA2) markers supposed to be expressed by nigral dopaminergic neurons^115^.

## Challenges and strategies to overcome issues related to stem cell therapy in neurodegenerative disorders

Stem cell therapy is now considered a treatment for various ailments; however, there are concerns related to the safety of the treatment. Fear has been expressed for the development of tumors post stem cell therapy since tumor cells are very much like stem cells expressing similar markers and divide indefinitely without going for differentiation. Thus, following up with the patients even years after the therapy is essential. Regarding this concern, a case has been presented where a patient treated with stem cell therapy for a rare neurodegenerative disorder, Ataxia telangiectasia, characterized by degeneration in the region controlling movement and speech, was presented with a glioneuronal tumor. HLA typing of the patient revealed the presence of both male and female cells in the tumor mass, which is suggestive of being sourced from two different donors. The development of tumors might be attributed to the impaired immune system in ataxia telangiectasia patients^116^. Though there was the slow growth of the tumor and cells were well differentiated, pointing toward the benign nature of the tumor, still the presentation of the tumors after fetal neural cell transplantation is worrying and needs work on the assessment of the safety of therapy and methods required for good differentiation of stem cells post-treatment. Donor-derived tumors might regress due to immune rejection. Thus, modulation of the immune system may be effective in patients undergoing stem cell therapy^117^. It is known that the exogenous expression of transcription factor Oct4 alone can transform adult mouse NSCs into PSCs. Such single-factor induced pluripotent cells encompass markers like ESCs and can be efficiently differentiated into neural cells, cardiomyocytes, and germ cells, along with the capability to form teratoma^118^. Considering the multifactorial nature of neurodegenerative disorders, non-tumorigenic undifferentiated stem cells are suggested. Pre-differentiated cells are often targeted and thus can address only one deficit, but the challenge of homing and dosing is still critical. Contrarily, undifferentiated cells might respond to various microenvironmental factors and might choose between differentiating or not and multiplying or not under a specific cellular microenvironment. Pre-differentiated stem cells might be unable to address the issues that physicians are unaware of^119^. ESCs might be teratogenic, and differentiated stem cells suffer a single fate. The solution to this problem is the usage of undifferentiated neural progenitor cells (NPCs), which are advanced versions of ESCs. At the same time, they have lost tumorigenicity and might still differentiate into different cells under the influence of microenvironmental factors according to the requirement. Though undifferentiated cells can migrate to the injury site, the homing and migration are low. For example, if the ailment is present in the cerebellum, injection of undifferentiated cells in the carotid artery or forebrain will have little or no effects^120^. Another challenge of stem cell therapy is the ethical issue where the usage of embryo blastocyst is considered as a murder of potential human life. Single-cell biopsy through micromanipulation and growing in culture has shown immense potential, and single blastomeres can be used to derive hESC lines without destroying the embryo and thus address the ethical issue^121^. Derivation of PSCs from adult tissue might address the problems associated with immune rejection and ethical concerns; however, identifying adult stem cells is still challenging^122^. Further studies are needed to determine whether other sources of cells, like dental pulp, can be used to generate induced pluripotent cells by expression Oct4 through nonretroviral-mediated expression^123^. Furthermore, the ability to form teratoma is a point of concern and needs to be addressed precisely^124,125^. Expression of Ngn2 inhibits cell division and neuron formation even in the presence of mitogen, while expression of Mash1 leads to cell division with differentiation into neurons. Thus, the fate of the neural progenitor cells might be determined using multiple transcription factors like Ngn2 and Mash1, and thus, the problem of teratoma formation may be prohibited^126^. We opine that such experiments should be conducted through well-designed clinical trials to ensure quality, safety, and ethical standards are met.

## Ethical issues

PSC lines are derived from the 5 to 7-day-old blastocyst. hESCs have always remained controversial since, from one perception if an embryo is implanted into a woman’s womb with appropriate hormonal phasing, it might develop into a live-born human being, and hESC leads to the destruction of the human embryo. According to the Orthodox church, the fertilized embryo results from the union of male and female nuclei. It unites the living energies of both souls, giving rise to new vital energy, and it exists immediately after the merger^127^. Thus, an embryo preserves the same rights and interests as an adult human or person. Taking the inner cell mass of blastocyst to obtain stem cells is, therefore, tantamount to murder. According to the Hindu religion also, life begins at the time of conception, and thus, destruction of the embryo is not permissible, and it is permissible only in incidences when it is threatening the mother’s life^128^. Removing inner cell mass from a 5 to 7-day-old blastocyst to generate human stem cells from this perception is tantamount to murder (Monitoring stem cell research. Washington, DC: The President’s Council on Bioethics)^129^. From another perception, the blastocyst is not more than a clump of cells (Research with human embryonic stem cells: ethical considerations)^130^. Keeping the ethics in mind, one might consider that frozen embryos, which are no longer required since the couple already has a child and no longer wants another one and is not willing to donate also to another couple, such embryos can be used in stem cell research to further save and improve lives instead of being discarded^131^. Following are some significant ethical concerns related to stem cell therapy

1. Usage of existing cell lines: Not all hESC cell lines are safe for transplantation due to the accumulation of mutations predisposing to cancer^132^. In the future, more embryo donors are needed to come forward so that more patients might receive human leukocyte antigen (HLA) matched stem cell transplants^133^.
2. Usage of frozen embryos: Several ethical concerns are raised in using frozen embryos in stem cell research, including the confidentiality and consent of the donor. After adopting the Nuremberg Code, informed consent is essential to research human subjects. Donors’ opinions differ regarding stem cell research; they allow the use of their embryos for infertility issues but not for stem cell works where the work might be used for commercial or patent purposes^134^. A waiver might be granted from consent if the biological material is deidentified and donors are unlocatable.
3. Consent and confidentiality of gamete donor: Embryos might be prepared from gametes by in vitro fertilization. People opine that informed consent is not required here since they have ceded their rights to use their reproductive material. A study conducted with oocyte donors concluded that 25% of women do not want their oocytes to be used in stem cell research^135^. The data relating to the gamete donor present in the computer files should be protected from hackers^136^.

## Ethical guidelines for stem cell therapy

There have been some ethical guidelines set to be followed by organizations from different countries to research stem cells. It includes mandatory testing for six major transmittable diseases (HIV-1 and 2, hepatitis B virus, hepatitis C virus, Treponema pallidum, human T-lymphotropic virus, and cytomegalovirus) and any genetic disorder. Apart from that, if any monetary benefit is obtained, it may be provided to the donor or society; however, the intellectual property rights do not belong to the donor. In the Indian context, establishing new ESC/iPSC lines is permissible. In contrast, research on human preimplantation embryos processed by in vitro fertilization (IVF)/intracytoplasmic sperm injection/somatic cell nuclear transfer to derive ESC lines is restricted^137^. It is nearly impossible to have an absolute consensus on stem cell research; however, it is possible to have a common opinion related to stem cell research. The examples are that women should neither be forced to overproduce eggs through hormonal injections nor paid hugely for egg donation that it becomes a business or exploitation. They should not be forced to donate eggs for research purposes. The privacy of donors should be strictly maintained, and the donors should be informed about the research related to stem cell therapy. In order to obtain ESCs, the embryos should not be cultured beyond the age of 14 days, the point when neural precursors are about to develop. Participants should have the right to decline to participate at any point in time. The research that involves human stem cell transplantation into animal models should be restricted and carefully monitored. When stem cell therapies translate into therapies, ethical standards must be followed, such as safe manufacturing and usage of viral pathogens or genetic disorders containing cell lines. It is also essential to discuss how much information is enough for a patient undergoing stem cell therapy, and critically ill patients should not be deceived with unproven cures^138^. In such context, guidelines given by competent authorities are essential statements with a commitment to ethical scientific research.

## Future directions and perspectives

Under experimental settings and in a few clinical studies, stem cell therapies have been shown to be effective in treating neurodegenerative disorders. Which type of stem cell will be most appropriate in treatment depends on the ailment to be addressed and the challenge. Despite having many challenges like limited success and lots of ethical issues, treatment of neurodegenerative disorders with stem cells is still an exciting option. Short-term usage of stem cells to provide the therapeutic factors to inhibit disease progression is advisable. Information like the delivery site, graft survival, and effectiveness of the therapy need to be addressed before initiating and continuing the clinical trials. A new cellular origin to generate stem cells and approaches to differentiate them at the desired site need to be used in combination. The use of ESCs and iPSCs might result in teratomas and/or cancer. On the other hand, MSCs, whether derived from bone marrow or adipocytes, have questionable survival and mode of action in the host^139^. Adipocyte-derived stem cells’ poor survival is partially due to a hypoxic environment in the transplanted area and might require specific scaffolds to obtain attachment, proliferation, and differentiation^140^. Similarly, the quality of human bone marrow-derived stem cells decreases after their detachment from the culture plate over a period of time, and the phenomenon is not understood yet clearly^141^. Thus, stem cell therapy might be clubbed with other strategies like nanotherapy to improve its efficacy and safety. Since the nanoparticles have high surface area-to-volume ratios, they offer tunable properties, and thus, nanomaterials can combat oxidative stress, scavenge abnormally aggregated proteins, and function as idea carriers for targeted stem cell therapy. The size, shape, charge, and surface modification influence stem cell differentiation through signaling pathways^142–144^. In the study of Dai *et al*.^145^, magnetic iron oxide nanoparticles (MIONs) were used as a differentiation modality. These nanoparticles offer the advantage of easy endocytosis. The differentiation might be induced by applying a magnetic field to differentiate mouse embryonic stem cells (mESCs) into nerve cells. A synergism was observed when chemical stimulation with retinoic acid was combined with the physical stimulation of MIONs in magnetic fields that induced a differentiation process significantly, and mature nerve cell marker gene expression level was found to increase by 60 folds. In conclusion, although various challenges persist, stem cell therapy offers a promising tool to fight neurodegenerative disorders, and it is believed that after putting more emphasis on experimentation and conducting more clinical trials, the picture will be clearer about the safety, efficacy, and utility of stem cell-based therapies for fighting neurodegeneration.

## Ethical approval

No ethical approval is required.

## Consent

No ethical committee approval and fully informed written consent is required.

## Sources of funding

Not applicable.

## Author contribution

R.K. and M.E.A.Z.: conceptualization, resources, data curation, writing – original draft, writing – review and editing, and supervision; P.G.: conceptualization, data curation, writing – original draft, writing – review and editing, and supervision; V.R., S.A.A.-H.: resources, data curation, writing – original draft, and editing.

## Conflicts of interest disclosure

All authors declare a lack of any conflict of interest or of competing interests.

## Research registration unique identifying number (UIN)

Not applicable.

## Guarantor

Rekha Khandia.

## Data availability statement

The data used in the present study is available publicly in PubMed and Pubmed Central, reference number 1-145.

## Provenance and peer review

The paper is invited by Dr Om Prakash Choudhary for the Special issue ‘Evolving trends in stem cell therapy.’
